# Urgent administration of antivenom following proven krait bites in Southeast Asia irrespective of neurotoxic symptoms

**DOI:** 10.1371/journal.pntd.0012079

**Published:** 2024-04-11

**Authors:** Joerg Blessmann, Benno Kreuels

**Affiliations:** 1 Research Group Snakebite envenoming, Department of Implementation Research, Bernhard Nocht Institute for Tropical Medicine, Hamburg, Germany; 2 Section for Tropical Medicine, I. Department of Medicine, University Medical Center, Hamburg, Germany; Rajarata University of Sri Lanka Faculty of Medicine and Allied Sciences, SRI LANKA

## Introduction

Neuromuscular paralysis because of snakebite envenoming is a life-threatening and common clinical syndrome caused by most elapids (family Elapidae) and few species of pit vipers and true vipers (family Viperidae) [1]. In Southeast Asia, cobras and kraits are responsible for most cases of neurotoxic envenoming. Two major neurotoxin classes, alpha-neurotoxins (α-NTs) and beta-neurotoxins (β-NTs), target the neuromuscular junction and cause flaccid muscle paralysis [2]. Beta-neurotoxins belong to the group I phospholipases A_2_ and act at the presynaptic motor nerve terminal. They are the major neurotoxins in krait venom and cause synaptic vesicle depletion with declining acetylcholine release and degeneration of the nerve terminal [3]. Phospholipases A_2_ have a molecular mass of 13 to 16 kDa which corresponds to 115 to 125 amino acids [2]. After subcutaneous injection of venom during the bite, toxins are resorbed from the interstitial space into the blood circulation either directly or through lymphatic vessels and leave the blood circulation to reach and act at the neuromuscular junction [4,5]. The small molecular size of neurotoxins facilitates the journey and most victims develop neurotoxic signs within 6 h after the bite [6]. Once neurotoxic signs are present and nerve terminals damaged, administration of antivenom will likely be ineffective. Here, we propose the administration of antivenom to all patients with a confirmed krait bite who present within the first 6 h after the bite, regardless of neurotoxic signs, and present arguments for this approach.

## Frequency of neurotoxic signs and duration of muscle paralysis after krait envenoming

Flaccid muscle paralysis of varying degrees is the major clinical symptom caused by β-NTs in krait venom. This becomes life-threatening when respiratory muscles are affected and immediate intubation and mechanical ventilation are necessary to save the victim’s life.

Krait bite envenoming often leads to prolonged failure of neuromuscular transmission caused by morphological damage to motor neuron endings. Animal studies in rats found full recovery of muscle strength 7 days after β-bungarotoxins were injected into the musculus soleus, without the use of antivenom [3].

In a retrospective case series of patients bitten by *Bungarus* species in Thailand, including 68 bites of *Bungarus candidus*, 9 of *Bungarus fasciatus*, and 1 of *Bungarus flaviceps*, 88.6% of the patients showed neurotoxic signs within the first 8 h after the bite and 76% required mechanical ventilation (Table 1). The median time between bite and antivenom administration was 5 h and the median time on the ventilator was 6 days [6].

**Table 1:** Neurotoxicity, respiratory failure, and ventilation period caused by *Bungarus* spp. in Thailand, Vietnam, and Taiwan.

Snake species	Country	No.	Time interval bite to AV	Neurotoxic signs (%)	Respiratory failure (%)	Ventilation period (days) median (range)	Reference
*B*. *candidus*	Thailand	68	50% < 5 h	88.6	79.4	6 (1–37)	Tongpoo et al. 2018 [6]
*B*. *fasciatus*		9	50% < 4 h		55.6	6 (1–15)	
*B*. *multicinctus*	Vietnam	27	7.5% < 6 h	100	85	2.3 (0–12)	Hung et al. 2010 [8]
*B*. *multicinctus*	Vietnam	60	n.a.*	93	87	8.2 (1–29)	Hung et al. 2009 [7]
*B*. *multicinctus*	Taiwan	44	86% < 4 h	75	27	(1.2–2.5) and (4.3–15.4) **	Yan-Chia Mao et al. 2017 [9]

*Patients were treated without antivenom.

**This refers to patients without (*n* = 7) and with (*n* = 5) complications during respirator treatment, respectively.

AV, Antivenom; B, Bungarus; No, number (of cases); h, hour; n.a., not applicable.

A retrospective study from Vietnam reported respiratory failure in 52 out of 60 (87%) patients with *Bungarus multicinctus* envenoming (Table 1). Patients were treated without antivenom with a mean time of 8.2 days on respirator and a case fatality rate of 7% [7]. In a second prospective study from Vietnam, 23 out of 27 cases (85%) of *B*. *multicinctus* envenoming developed respiratory failure. Patients in this study were treated with antivenom and the time on respirator was significantly shorter at 2.3 days and no death was reported [8]. A significantly lower number of respiratory failures with 12 out of 44 cases (27%) after *B*. *multicinctus* envenoming was reported from Taiwan [9]. In this study, the time interval between bite and antivenom administration was significantly shorter with 86% of patients receiving antivenom within the first 4 h after the bite compared to only 7.5% of patients receiving antivenom within the first 6 h in Vietnam (Table 1).

Timely administration of antivenom seems to reduce the duration of neurotoxicity and the number of patients developing more severe neurotoxicity with respiratory failure. Shortening the time interval between bite and administration of antivenom presumably plays a crucial role and will likely reduce the number of patients developing respiratory failure and time on a respirator, leading to fewer complications with lower mortality.

## Limitations of the available data

All data presented in Table 1 refer to snakebite patients seeking medical care in hospitals [6–9]. This might be a selection of more severe cases, with a higher percentage of neurotoxic signs and respiratory failure. However, health systems in Thailand and particularly in Taiwan are well developed and expenditures for treatment of snakebite envenoming are reimbursed by the universal health coverage system. In Thailand, access to life-saving snake antivenom significantly improved since the implementation of the Thai national antidote program in 2010 [10]. Hence, it is likely that most snakebite patients seek medical care in hospitals, particularly those after bites of venomous species. Furthermore, data were collected retrospectively except in one study from Vietnam [8].

## Side effects of horse-derived F(ab)_2_ antivenom from the Thai Red Cross, Bangkok, Thailand

The Thai Red Cross in Bangkok, Thailand is the main producer of antivenom in Southeast Asia and the only producer of antivenom against *B*. *candidus* in the region. These antivenoms are used in Thailand and exported to neighboring countries, namely to Laos, Vietnam, Cambodia, Indonesia, and Malaysia. A recent publication from Thailand showed that 154 out of 684 (22.5%) developed early adverse reactions (EARs) and 30 (4.4%) an anaphylactic shock. Under close monitoring during antivenom administration and appropriate treatment of EARs, no death was reported [11].

## Significance for clinical management of neurotoxic envenoming in Southeast Asia

The World Health Organization’s Guideline for management of snakebites in Southeast Asia recommends: “Antivenom should be given only to patients in whom its benefits are considered likely to exceed its risks. Since antivenom is costly and often in limited supply, it should not be used indiscriminately. The risk of reactions should always be taken into consideration.” In the particular case of neurotoxic envenoming, antivenom administration is recommended only if neurotoxic signs are present [12]. However, the published case series from Vietnam and Thailand indicate that a very high proportion of patients develop signs of neurotoxicity with respiratory failure after bites of *Bungarus* species. The long persistence of muscle paralysis leads to prolonged ventilation times with a high risk for complications. In these cases, it seems plausible that earlier administration of antivenom, before signs of neurotoxicity appear, could reduce the proportion of patients who develop severe envenoming by neutralization of neurotoxins within the blood circulation. A clinical trial to investigate the early administration of antivenom before neurotoxic signs occur compared to current standard of care would be desirable. However, kraits are night active snakes and bites are rare. Hence, enrollment of sufficient cases would be a challenge. In contrast to the WHO recommendation, the Queen Saovabha Memorial Institute in Bangkok and the Thai Red Cross Society already recommend antivenom administration to all patients with confirmed and suspected krait bites regardless of neurotoxic signs, as published in their 2013 Manual of Practical Management of Snakebites and Animal Toxin Injury [13]. The recommended dose of the Thai Red Cross is 5 vials of monovalent Malayan or Banded Krait antivenom or 5 vials of Polyvalent neurotoxic antivenom. Although the rate of early adverse reactions is high and can be life-threatening, these reactions are well controlled if appropriate monitoring is in place and adrenaline at hand [11]. In clinical practice, we propose that clinicians should consider administering antivenom to all patients presenting with a confirmed krait bite within the first 6 h after the bite regardless of neurotoxic signs. A confirmed krait bite means that either the dead snake or a picture of the culprit snake is available for correct identification. The 6-h time window after the bite is an important precondition since the majority of patients develop neurotoxic signs during this time. In these cases, the potential benefit likely outweighs the risk of potential side effects of the antivenom. For patients who present without neurotoxic signs more than 6 h after the bite, the risks would likely outweigh the benefits and antivenom should only be given in the presence of neurotoxic symptoms. As mentioned above, kraits are night active snakes and the number of krait bites is low. In Laos, krait bites account for approximately 5% of all snakebites [14]. The number of patients who present very early after the bite without neurotoxic symptoms is even lower, particularly in regions with less developed health systems, high out-of-pocket payment, low health literacy, and poor road infrastructure. However, as the example of Taiwan showed people will arrive earlier if these indicators improve [9]. Confirmation of the snake being a krait is another challenge. However, based on our experience in Laos approximately 20% of patients bring the snake to the hospital and an increasing number of patients provides a picture or video for correct identification of the culprit snakes taken with a smartphone [14]. Six different species of the genus *Bungarus* are present in Southeast Asia, namely *B*. *candidus*, *B*. *(multicinctus) wanghaotingi*, *B*. *fasciatus*, *B*. *slowinskii*, *B*. *flaviceps*, and recently described *Bungarus sagittatus*. *B*. *candidus*, *B*. *fasciatus*, and *B*. *(multicinctus) wanghaotingi* seem to be responsible for the majority of bites in Southeast Asia [6–8]. *B*. *candidus* and *B*. *fasciatus* are found in Thailand, Laos, Vietnam, Cambodia, Malaysia, Indonesia, and Myanmar and *B*. *(multicinctus) wanghaotingi* in Thailand, Laos, Vietnam, Myanmar, and Malaysia [15]. Antivenom against *B*. *candidus* and *B*. *fasciatus* is produced by the Queen Saovabha Memorial Institute, Bangkok, Thailand and against *Bungarus fasciatus* by Biofarma in Indonesia [16]. Currently, there is no antivenom against *Bungarus (multicinctus) wanghaotingi* available in Southeast Asia.

The number of patients to whom this recommendation may apply in practice and the overall impact on morbidity and mortality of snakebites is likely small. However, for the affected individuals and their families a possibly shortened time on mechanical ventilation with a lower risk for complication make a huge difference. Further literature research needs to be done whether the recommendation would be applicable to other regions as well, particularly for South Asia, where different and diverse *Bungarus* species are present.

## Conclusions

The vast majority of patients presenting to hospitals in Southeast Asia with krait bite envenoming develop neurotoxic signs and respiratory failure. Once paralysis has occurred, resistance against antivenom treatment is likely and there is a risk of long-term ventilation. In our opinion, this justifies the administration of antivenom to all patients with a confirmed krait bite within the first 6 h after the bite, regardless of neurotoxic signs. It applies at least to Southeast Asia, where *Bungarus candidus*, *Bungarus fasciatus*, and *Bungarus (multicinctus) wanghaotingi* are the main culprit snakes.
